# Cytoglobin regulates blood pressure and vascular tone through nitric oxide metabolism in the vascular wall

**DOI:** 10.1038/ncomms14807

**Published:** 2017-04-10

**Authors:** Xiaoping Liu, Mohamed A. El-Mahdy, James Boslett, Saradhadevi Varadharaj, Craig Hemann, Tamer M. Abdelghany, Raed S. Ismail, Sean C. Little, Danlei Zhou, Le Thi Thanh Thuy, Norifumi Kawada, Jay L. Zweier

**Affiliations:** 1 Davis Heart and Lung Research Institute and Division of Cardiovascular Medicine, Department of Internal Medicine, College of Medicine, The Ohio State University, Columbus, Ohio 43210, USA; 2 Department of Hepatology, Graduate School of Medicine, Osaka City University, Asahimachi 1-4-3, Abenoku, Osaka 545-8585, Japan

## Abstract

The identity of the specific nitric oxide dioxygenase (NOD) that serves as the main *in vivo* regulator of O_2_-dependent NO degradation in smooth muscle remains elusive. Cytoglobin (Cygb) is a recently discovered globin expressed in fibroblasts and smooth muscle cells with unknown function. Cygb, coupled with a cellular reducing system, efficiently regulates the rate of NO consumption by metabolizing NO in an O_2_-dependent manner with decreased NO consumption in physiological hypoxia. Here we show that Cygb is a major regulator of NO degradation and cardiovascular tone. Knockout of *Cygb* greatly prolongs NO decay, increases vascular relaxation, and lowers blood pressure and systemic vascular resistance. We further demonstrate that downregulation of *Cygb* prevents angiotensin-mediated hypertension. Thus, Cygb has a critical role in the regulation of vascular tone and disease. We suggest that modulation of the expression and NOD activity of Cygb represents a strategy for the treatment of cardiovascular disease.

## Abstract (web summary)

The gaseous signalling molecule nitric oxide regulates vascular tone. Here, the authors show that nitric oxide is degraded by the enzyme cytoglobin in the vascular wall, and that mice lacking cytoglobin have reduced blood pressure and are less sensitive to angiotensin-mediated hypertension.

Endothelium-derived relaxing factor, identified as nitric oxide (NO), is a key mediator regulating vascular tone and blood pressure (BP)^1^,^2^. NO mediates vascular relaxation through binding to and activation of soluble guanylate cyclase (sGC) in the smooth muscle of vessels^3^. Vascular NO levels are controlled by both the rate of NO generation and the rate of NO metabolism. While NO is synthesized by a specific well characterized NO synthase in the endothelium (eNOS), the process of NO degradation and metabolism in the vascular wall is poorly understood^4^,^5^,^6^. It is hypothesized that NO degradation in the vessel wall is mediated by an O_2_-dependent NO dioxygenase (NOD) such as myoglobin (Mb), haemoglobin-α (Hb-α) or cytoglobin (Cygb) that oxidizes NO to nitrate^7^,^8^,^9^,^10^,^11^,^12^,^13^,^14^. However, the identity of the specific NOD that functions as the main *in vivo* regulator of O_2_-dependent NO degradation in smooth muscle is still unknown. Additional questions also remain regarding the identity of the cellular reducing system that couples with this NOD to regulate the rate of NO consumption in vascular smooth muscle^12^,^15^.

Each member of the globin family of proteins has a unique pattern of cellular expression and localization. Tetrameric Hb is mainly located in red blood cells^16^, Mb is mainly located in cardiac and skeletal muscle^17^, neuroglobin is mainly present in neurons^18^,^19^ and monomeric Hb-α has recently been discovered in the myoendothelial junction of resistance vessels^14^. Cygb has been found predominantly in fibroblasts and in the vascular wall^13^,^14^,^20^. The concentration of Hb-associated haem in blood approaches 8 mM, and the concentration of Mb in heart and skeletal muscle is several hundred micromolar or higher. At such high globin concentrations, the rate of NO consumption in blood, heart and skeletal muscles is very rapid^21^,^22^,^23^,^24^,^25^. Unlike Hb and Mb, the Cygb concentration in cells where it is expressed, such as smooth muscle, is in the micromolar range^26^,^27^. At this concentration, Cygb could play a role in regulating NO concentrations in the smooth muscle of the vessel wall, where NO levels are of critical importance for activation of sGC, which in turn regulates vascular tone^1^,^3^.

In cells and tissues where Mb is highly abundant such as in cardiac muscle, Mb has been reported to function as a potent NOD to reduce cytosolic NO concentrations^22^,^28^. This process was shown to be crucial for the breakdown of NO in cardiac muscle and to regulate the dose–response of the effects of NO on the heart. Hearts with genetic knockout of Mb were more sensitive to infused NO with increased cardiac depression and vasodilation. This NOD function was further hypothesized to protect myocyte cytochromes against increases in NO levels^22^. However, Mb has been reported to be either absent from vascular smooth muscle or present in only trace amounts, leading to the concept that there must be another globin with the primary function of regulating NO degradation in vascular smooth muscle^13^,^15^,^29^.

Recently, it has been reported that Hb-α is expressed in endothelial cells and enriched at the myoendothelial junction in small arteries and arterioles, where it can serve to regulate NO flux out of the endothelial cell to the vascular smooth muscle^14^,^30^. Endothelial cytochrome b5 reductase 3 was further reported to regulate this process through the reduction of the haem iron of Hb-α, and genetic and pharmacological inhibition of cytochrome b5 reductase 3 was found to enhance NO bioactivity in small vessels. Thus, Hb-α has been reported to play a critical role as a NOD located at the myoendothelial junction where it can serve to regulate NO efflux from the endothelium.

Questions remain regarding the process of NO metabolism in vascular smooth muscle and how this regulates vascular tone. While Cygb is expressed in smooth muscle cells (SMCs), its function has not yet been elucidated^31^,^32^,^33^,^34^. When coupled with suitable cellular reducing systems, such as ascorbate or cytochrome b5 reductase/cytochrome b5/NADH, Cygb has been demonstrated to function as a NOD, efficiently regulating the rate of O_2_-dependent NO consumption^12^,^15^. Cygb uniquely metabolizes NO in a highly O_2_-dependent manner with decreased NO consumption in physiological hypoxia^35^,^36^,^37^. As such, one can hypothesize that regulation of the expression level or NOD function of Cygb could modulate basal vascular tone and blood pressure.

In this study, we demonstrate in cellular, isolated vessel and *in vivo* models, that Cygb is a highly efficient NOD, and serves as a major regulator of NO degradation and cardiovascular tone in the vascular wall. Both ascorbate and cytochrome b5 reductase/cytochrome b5/NADH serve as effective reducing systems for Cygb, with the latter serving as the major reducing system in SMCs. Knockout of *Cygb* greatly prolongs NO decay, increases vascular relaxation and lowers blood pressure and systemic vascular resistance (SVR) with increased tissue perfusion. Furthermore, it was observed that downregulation of *Cygb* can prevent angiotensin-mediated hypertension. Thus, Cygb is shown to have a critical role in the regulation of vascular tone and prevention of disease.

## Results

### Globin expression level, reduction and NO consumption

In order to assess the importance of a given globin protein in the metabolism and consumption of NO in smooth muscle, it is necessary to determine at what level it is expressed and what its rates of reduction and NO consumption are. Therefore, initial experiments were performed in smooth muscle cells (SMC) first to measure the expression levels of the globin proteins Cygb, Mb and Hb-α that have been reported to have important NOD function. These experiments were then followed by spectrophotometric measurements of the reduction rates and electrochemical measurements of the rates of NO consumption by each globin in the presence of ascorbate or an enzymatic reducing system.

**Figure 1:**
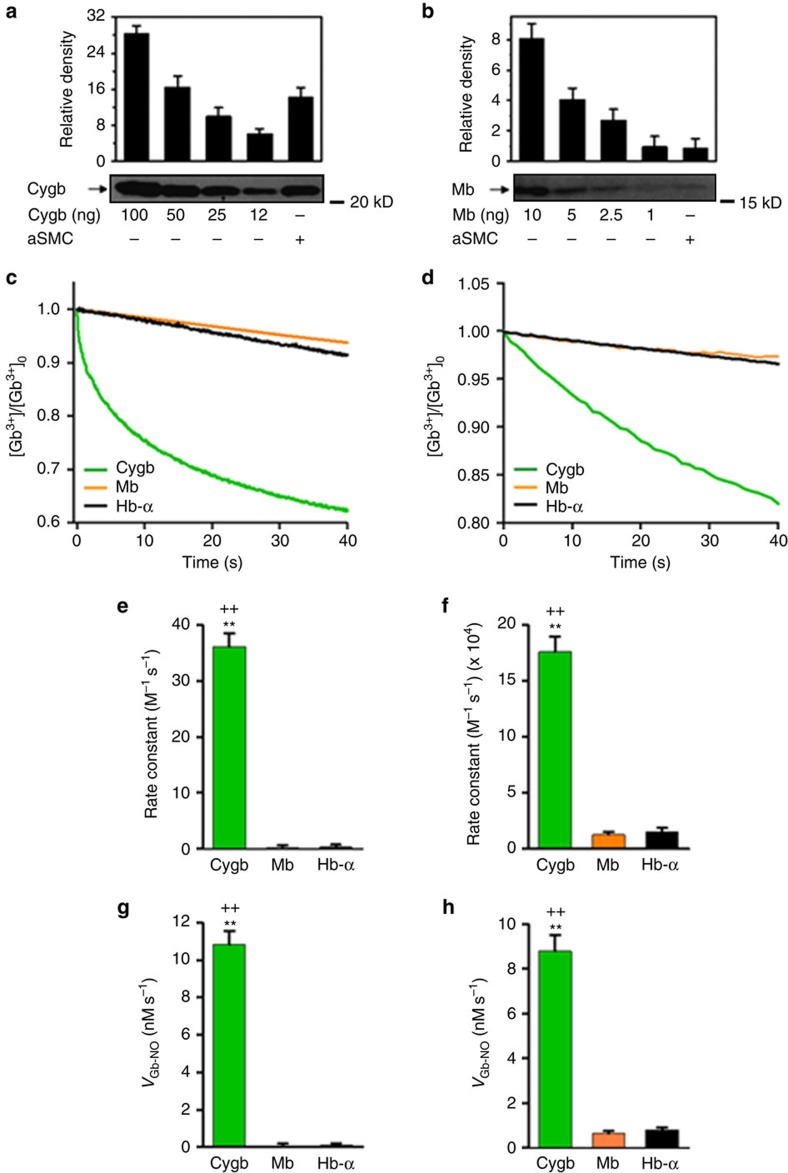
Expression of globin proteins, reduction rates and rates of NO consumption of Cygb, Mb and Hb-α. Level of Cygb (**a**) and Mb (**b**) in human aortic smooth muscle cells (aSMCs) measured by quantitative immunoblotting. These results indicate that there is ∼45 ng of Cygb and ∼1 ng of Mb in 10^6^ aSMCs. Hb-α was not detectable with a level >200-fold below that of Cygb. Assuming a cell volume of 400 μm^3^ (ref. 11), the intracellular concentration of Cygb is estimated at ∼5.3 μM and that of Mb at ∼0.13 μM and Hb-α<0.03 μM. Positions of nearest molecular weight markers are shown. Reduction of globins by 10 mM Asc (**c**) or b5R (30 nM)/b5 (0.5 μM)/NADH (100 μM) (**d**). Measured rate constants of Cygb, Mb and Hb-α reduction by 10 mM Asc (**e**). Measured rate constants of Cygb, Mb and Hb-α reduction by b5 reductase (30 nM)/b5 (0.5 μM)/NADH (100 μM) (**f**). Calculated rate of NO consumption by Cygb, Mb and Hb-α in the presence of 1 μM globin and 0.3 mM Asc (**g**). Calculated rate of NO consumption by Cygb, Mb and Hb-α in the presence of 1 μM globin and 50 nM b5 reductase with excess b5 and NADH (**h**). The calculation was based on the equation in Supplementary Fig. 1, also for more detail see ref. 61. Error bars: mean±s.e.m., *n*=3 per group; **P<0.01 for Cygb versus Mb, ^++^*P*<0.01 for Cygb versus Hb-α; *P* values determined using a two-tailed *t*-test.

Quantitative immunoblotting was performed comparing the level of each globin from SMC homogenates to a series of purified protein standards of known concentration for each globin. From this quantitative immunoblotting, we observe that Cygb is the most abundant globin in aortic smooth muscle cells (aSMC) (Fig. 1a,b). We measure that the concentration of Cygb is ∼5 μM, while Mb levels are over 40-fold lower. Hb-α is trace or undetectable with levels >200-fold below those of Cygb.

Since the process of globin reduction is the rate-limiting step for NOD activity^37^, it is important to characterize the relative kinetics of globin reduction. Cygb has a uniquely fast reduction rate^15^,^36^. We observe that the reduction rate of Cygb is ∼10 times faster than that of other globins such as Mb or Hb-α when reductase systems (such as cytochrome b5 reductase/cytochrome b5/NADH) are used, and several hundred times greater when ascorbate (Asc) is the reductant (Fig. 1c–f). This leads to a 10- to 100-fold more rapid rate of NO consumption by Cygb than other globins (Fig. 1g,h). Thus, based on its relatively high expression level and its high NOD activity, Cygb would be predicted to be the major pathway of NO degradation in SMCs.

### Measurement of the product of NO consumption by Cygb

**Figure 2:**
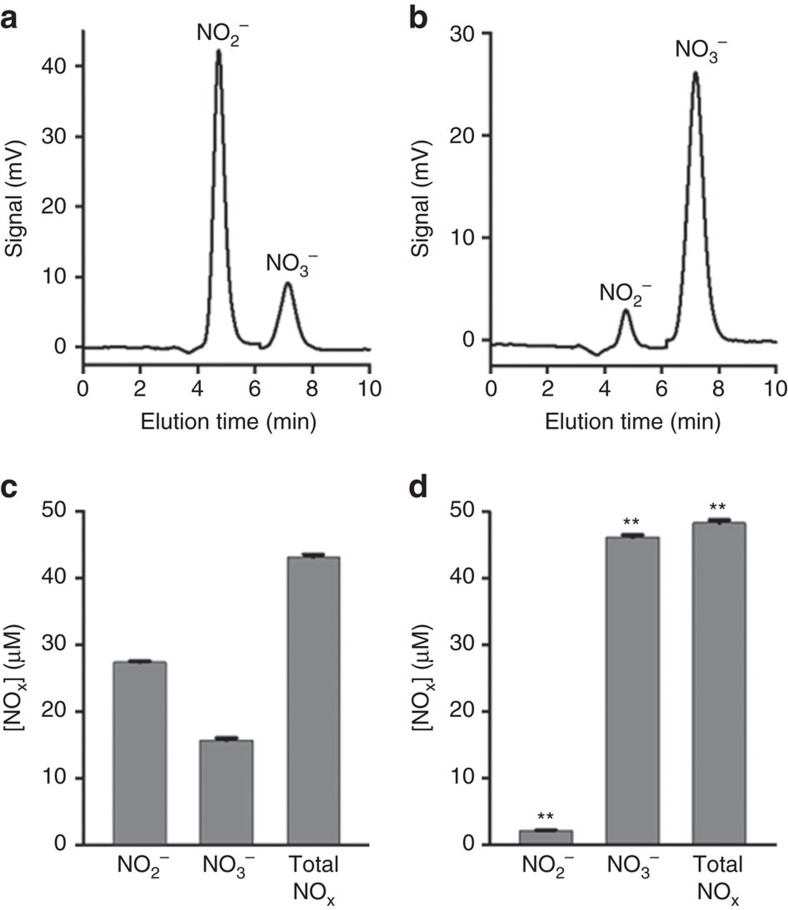
NO dioxygenase activity of cytoglobin. Nitrite and nitrate measurements of DEA-NONOate-treated samples without Cygb (**a**,**c**) and with the addition of 2 μM Cygb (**b**,**d**). Example elution profiles from the ENO-20 NOx analyzer for control (**a**) and Cygb (**b**) samples, with the corresponding concentrations in **c**,**d**, respectively. Addition of Cygb results in 96% of NOx as nitrate versus 36% in control. Nitrite comprises 64% of NOx in control but decreases to 4% in the Cygb samples. Error bars: mean±s.e.m., *n*=4 per group; **P<0.01 for Cygb versus control with no Cygb; *P* values determined using a two-tailed *t*-test.

In order to confirm that Cygb consumes NO through the process of NO dioxygenation where nitrate is the product, we assayed for the amounts of the NO degradation products nitrite and nitrate in the presence and absence of Cygb using an HPLC-based NOx analyzer. In the absence of Cygb, the major NO degradation product is nitrite, with only small amounts of nitrate detected (Fig. 2a,c), while in the presence of Cygb almost exclusively nitrate formation is observed (Fig. 2b,d). Thus, Cygb functions as a NOD with conversion of NO to nitrate.

### Role of Cygb in NO metabolism in vascular smooth muscle

**Figure 3:**
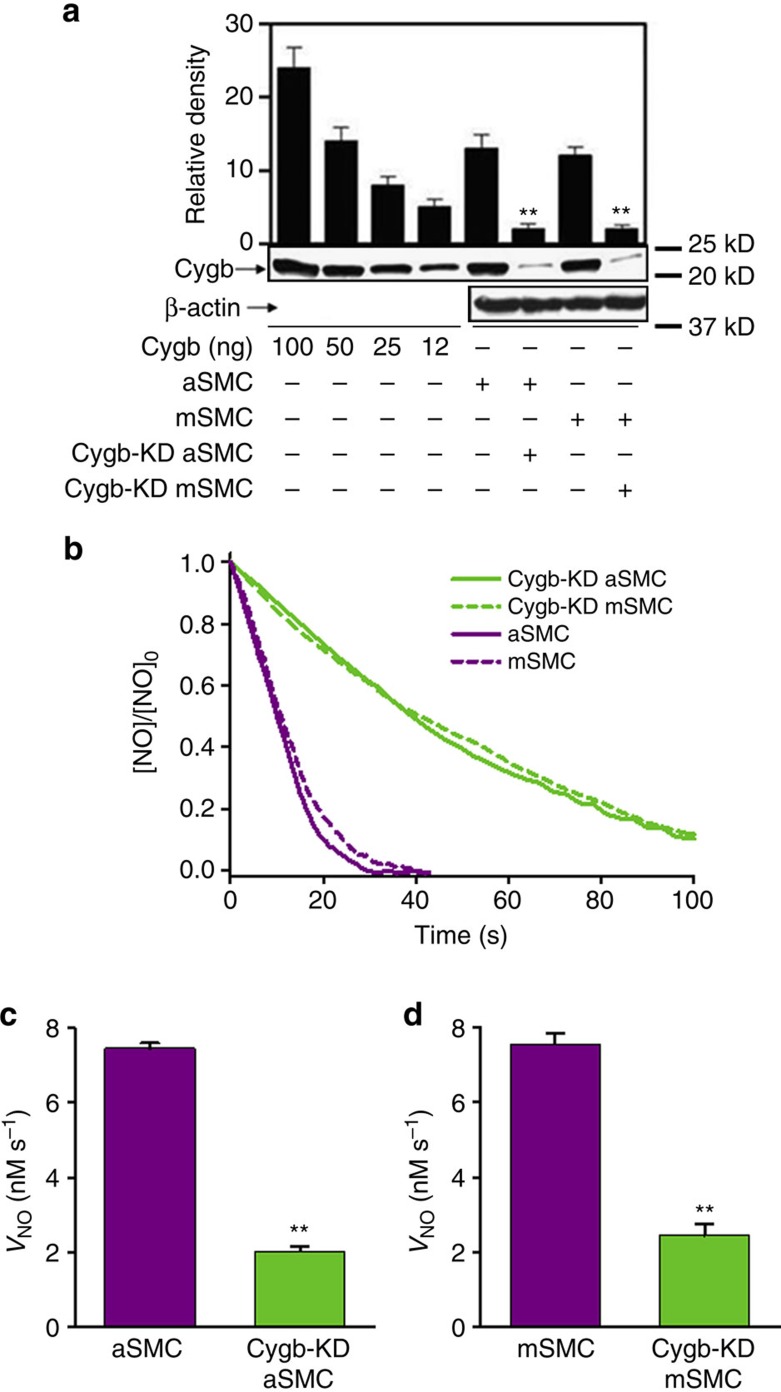
Knockdown of Cygb in rat aortic and mesenteric smooth muscle cells (SMCs). (**a**) Level of Cygb in rat aortic SMCs (aSMCs) and rat mesenteric SMCs (mSMCs) was estimated by quantitative immunoblotting. The first four bands are pure Cygb at four different amounts. Bands 5 and 6 are total cellular proteins from rat aSMCs and the related Cygb siRNA-treated cells (*Cygb*-KD aSMC), respectively; and bands 7 and 8 are rat mSMCs and the related Cygb siRNA-treated cells (*Cygb*-KD mSMC), respectively. Positions of nearest molecular weight markers are shown. (**b**) Plots of the rate of NO decay by 7 × 10^6^ SMCs per ml (violet solid line: aSMC, violet dashed line: mSMC) and 7 × 10^6^ *Cygb*-KD SMCs per ml (green solid line: *Cygb*-KD aSMC, green dashed line: *Cygb*-KD mSMC) versus time after NO was injected into the solution to achieve an initial concentration of 0.5 μM. Means and standard errors of the rate of NO decay by SMCs and *Cygb*-KD SMCs from aorta (**c**) and mesenteric artery (**d**). Error bars: mean±s.e.m., *n*=3–5 per group, **P<0.01 control SMCs versus *Cygb*-KD SMCs; *P* values determined using a two-tailed *t*-test.

In order to determine the role of Cygb on NO metabolism in smooth muscle of conduit and resistance vessels, we measured the rate of NO consumption in rat aSMCs and mesenteric SMCs (mSMC), as well as matched cells treated with Cygb-siRNA to knockdown Cygb expression. Cygb levels were evaluated by immunoblotting (Fig. 3a). The first four bands correspond to standard amounts of pure Cygb. Bands 5 and 6 are from homogenates obtained from control aSMCs and matched Cygb siRNA-treated cells; bands 7 and 8 are control mSMCs and matched Cygb siRNA-treated cells. Cygb levels were ∼80% depleted in these Cygb siRNA-treated SMCs.

With knockdown of Cygb, the rate of NO consumption measured by electrochemical NO sensor in aSMCs or mSMCs was decreased by ∼70–75% (Fig. 3b–d). With Cygb-siRNA treatment, ∼20% of basal Cygb levels remain (Fig. 3a), which suggests that the process of NO consumption in these SMCs is largely Cygb dependent, with <12% through other pathways.

### Role of b5R in NO metabolism in vascular smooth muscle

**Figure 4:**
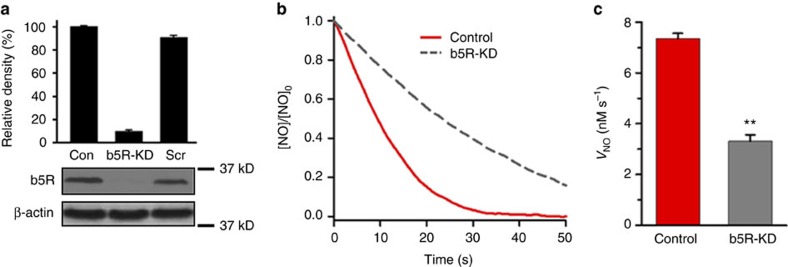
Effect of b5R on the rate of NO consumption by aortic smooth muscle cells. Relative amount of b5R in rat aortic vascular SMCs was estimated by quantitative immunoblotting before and after knockdown of b5R. Positions of nearest molecular weight markers are shown. (**a**) NO consumption by 7 × 10^6^ control SMCs per ml and by 7 × 10^6^ *b5R*-KD SMCs per ml. (**b**) NO was injected into the solution to achieve an initial concentration of 0.5 μM. Means and standard errors of the rate of NO decay by 7 × 10^6^ SMCs per ml and 7 × 10^6^ *b5R*-KD SMCs per ml (**c**). Error bars: mean±s.e.m., *n*=6 per group, **P<0.01 control SMCs versus *b5R*-KD SMCs; *P* values determined using a two-tailed *t*-test.

Questions remain regarding which cellular reducing system is involved in the process of Cygb reduction and NO dioxygenation. Since it is thought that cytochrome b5 reductase 3 (b5R) is of particular importance as a globin reductase, experiments were performed in vascular SMCs with b5R-siRNA treatment to knockdown b5R expression. From immunoblotting experiments as shown in Fig. 4a, the b5R-siRNA was highly effective in decreasing b5R expression with ∼90% decrease seen while control scrambled siRNA had no effect. With this decrease in b5R expression, the rate of NO consumption was decreased by ∼60% (Fig. 4b,c). Based on the 90% efficiency of knockdown of b5R, this suggests that at least 67% of the NO consumption in the SMCs is b5R dependent.

### Globin expression levels and localization in vessels

**Figure 5:**
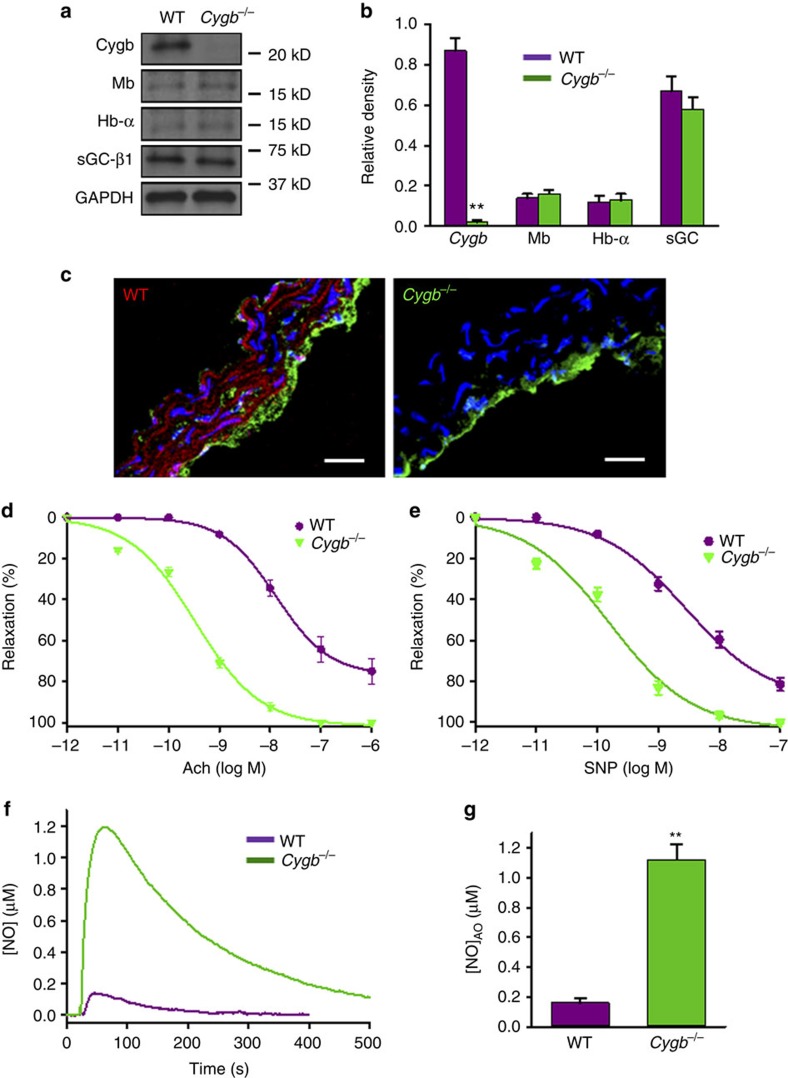
Expression of proteins in the NO-sGC pathway, NO-dependent vasodilation and vascular NO metabolism rate in WT and *Cygb*^−*/*−^ mice. Expression of Cygb, Mb, Hb-α, sGC-β1 and GAPDH in aortas of WT and *Cygb*^−*/*−^ mice. Positions of nearest molecular weight markers are shown (**a**) and their relative band density (**b**). Of note, to be able to detect Mb and Hb-α, 2.5-fold higher levels of homogenate protein were used compared to Cygb. Cygb staining (red) in WT and *Cygb*^−*/*−^ aortic sections with endothelium staining for eNOS (green). Scale bars, 50 μm (**c**). The phenylephrine-precontracted aorta of *Cygb*^−*/*−^ mice was much more sensitive to both Ach-induced relaxation (**d**) and SNP-induced relaxation (**e**). The EC_50_ for Ach-induced relaxation of *Cygb*^−*/*−^ and WT mouse aortas is 0.33 and 13 nM, respectively. EC_50_ for SNP-induced relaxation of *Cygb*^−*/*−^ and WT mouse aortas is 0.15 and 3.0 nM, respectively (**d**,**e**). Each data point in **d**,**e** represents the mean of six independent experiments. The measured NO concentration at the electrode surface after NO diffuses across the aortic wall, which is proportional to the NO flux through the vessel wall, of WT (violet) and *Cygb*^−*/*−^ (green) mice is shown (**f**). The measurement method for NO concentration was as previously described^35^. NO was injected into the solution to achieve an initial concentration of 3 μM. The peaks observed correspond to the maximum NO flux. The mean and standard errors of the peak NO concentration detected (**g**). Error bars: mean±s.e.m., *n*=5 per group, **P<0.01 WT versus *Cygb*^−*/*−^; *P* values determined using a two-tailed *t*-test.

To further evaluate the role of Cygb in vascular NO degradation, the levels and location of Cygb expression were measured in vessels of wild-type (WT) and *Cygb*^−*/*−^ mice. Similar to the results in isolated SMCs, immunoblotting of WT aortic homogenates demonstrated that Cygb expression was by far the highest among all globins tested, while in *Cygb*^−*/*−^ vessels there was no detectable Cygb and the expression of sGC, Mb and Hb-*α* were not significantly different in *Cygb*^−*/*−^ and WT aorta (Fig. 5a,b). Immunohistochemistry demonstrated that in WT mice, Cygb is highly expressed in SMCs (red staining) but is not present in the endothelium (green staining of eNOS as an endothelial marker). In contrast, Cygb is not detected anywhere in the vascular wall or endothelium of *Cygb*^−*/*−^ mice (Fig. 5c). Thus, Cygb is the major globin expressed in vascular smooth muscle and is not present in the endothelium.

### Vasodilatory function and NO degradation in vessels

To examine the effect of Cygb on vascular tone, we measured vasodilatory response of isolated aortic segments to endogenous and exogenous NO. Phenylephrine-precontracted aortas of *Cygb*^−*/*−^ and WT mice were studied. The *Cygb*^−*/*−^ vessels were much more sensitive to either the endothelium-dependent agonist acetylcholine (ACh) or the endothelium-independent NO donor nitroprusside, with a marked shift to the left in the vasodilation–response curves observed for *Cygb*^−*/*−^ compared to WT, with 39-fold lower (13–0.33 nM Ach) or 20-fold lower (3.0–0.15 nM nitroprusside) values seen for 50% relaxation, respectively (Fig. 5d,e). To determine if the enhancement of vasodilation in *Cygb*^−*/*−^ vessels is due to a lower rate of NO metabolism in the vessel wall, we measured the NO diffusion across the vascular wall of aortas from *Cygb*^−*/*−^ and WT mice using an NO electrode^35^,^38^ (Supplementary Fig. 2). To prevent interference from endothelium-derived NO, the endothelium was removed by gently rubbing the endothelial surface of the opened aortic segment^39^,^40^. The NOS inhibitor L-NAME (1 mM) was also added to inhibit NO generation from any remaining NO synthases. The measured peak NO flux across the aortic wall of *Cygb*^−*/*−^ mice was >6 times higher than that of WT (Fig. 5f,g). Thus, Cygb regulates endothelium-mediated vasodilation and vascular tone through its metabolism of NO.

### Role of Cygb on BP, cardiac function, vascular tone and cGMP

**Figure 6:**
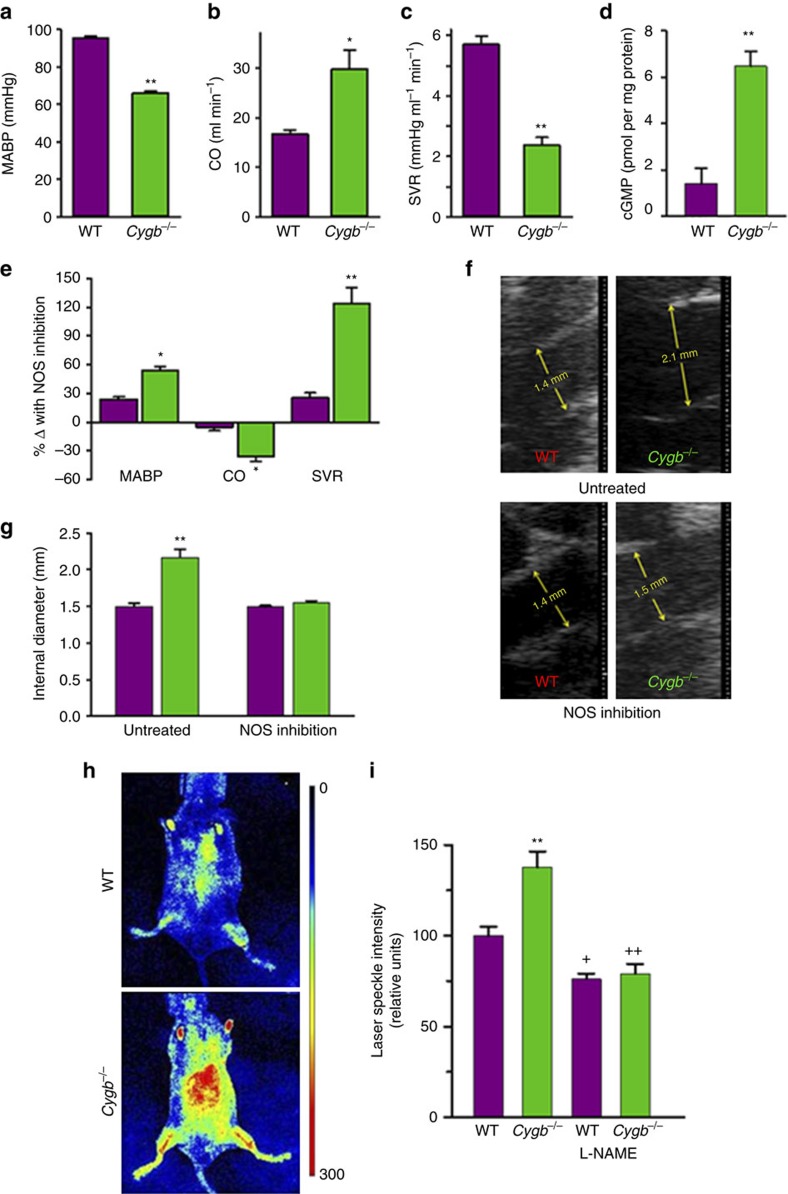
Cardiovascular function, perfusion, and NO signalling in WT and *Cygb*^−*/*−^ mice. MABP (**a**), CO (**b**), SVR (**c**) *n*=10 per group, cGMP levels in aortas of WT and *Cygb*^−*/*−^ mice (**d**) *n*=4 per group, and percent change in cardiovascular functions after NOS inhibition (**e**), *n*=7 per group. Inner diameter of aortas in WT mice and *Cygb*^−*/*−^ mice before and after treatment with NOS inhibitor L-NAME (**f**), and the mean and standard error of inner diameters of aortas (**g**) *n*=5 per group. *P<0.05, **P<0.01 WT versus *Cygb*^−*/*−^. Perfusion maps show higher baseline perfusion (red) in *Cygb*^−/−^ mice compared to WT mice as measured on the ventral side of the mouse. Colour scale (arbitrary units) is defined as highest perfusion in red and lowest perfusion in blue (**h**). In C*ygb*^−*/*−^ mice, ∼40% higher tissue perfusion was seen compared to WT mice and L-NAME treatment reversed this higher perfusion in *Cygb*^−*/*−^ mice to values similar to those in L-NAME-treated WT mice (**i**), *n*=4–12 per group. **P<0.01 WT versus *Cygb*^−*/*−^, ^+^*P*<0.05, ^++^*P*<0.01 WT or *Cygb*^−*/*−^ versus L-NAME-treated. Error bars: mean±s.e.m.; *P* values determined using a two-tailed *t*-test.

Further measurements were performed to determine the role of Cygb on *in vivo* BP, cardiac function and vascular tone. The mean arterial BP of *Cygb*^−*/*−^ mice was 30% lower with values of 65.3±1.9 mmHg for *Cygb*^−*/*−^ and 93.7±1.5 mmHg for WT (Fig. 6a). By echocardiography, cardiac output (CO) was increased by 68% in *Cygb*^−*/*−^ mice compared to WT (Fig. 6b). SVR of *Cygb*^−*/*−^ mice was decreased by 54% from that in WT mice (Fig. 6c). cGMP levels in *Cygb*^−*/*−^ aortas were five-fold higher than those of WT (Fig. 6d). In addition, the ascending aorta was clearly dilated in *Cygb*^−*/*−^ mice compared to WT mice with 47% increase in diameter (from 1.5±0.05 mm to 2.2±0.1 mm) (Fig. 6f,g). Thus, *Cygb* knockout results in increased activation of sGC with elevated levels of cGMP, causing marked vasodilation with lower BP and SVR that, in turn, triggers a compensatory elevation in CO.

### Role of NOS-derived NO in vascular relaxation

In order to further confirm that the diminished tone and enhanced vascular relaxation in *Cygb*^−*/*−^ mice were due to NOS-derived NO, mice were administered the NOS inhibitor L-NAME. L-NAME exerted large effects on the cardiovascular function of *Cygb*^−*/*−^ mice, reversing the low mean arterial BP (MABP) and SVR values as well as the elevated CO to values close to WT, while in WT lesser effects were seen as expected based on the lower levels of cGMP present (Fig. 6d,e). Cardiac echo imaging revealed that the aorta was dilated in *Cygb*^−*/*−^ mice compared to WT. After L-NAME treatment, the aortic dilation in *Cygb*^−*/*−^ relative to WT was also reversed (Fig. 6f,g). In order to obtain further data on microvascular function, perfusion imaging was performed on WT and *Cygb*^−*/*−^ mice. In *Cygb*^−*/*−^ mice, ∼40% increase in tissue perfusion was seen compared to WT mice. L-NAME treatment reversed this relative increase in tissue perfusion in *Cygb*^−*/−*^ mice to values similar to those in L-NAME-treated WT mice (Fig. 6h,i). Thus, inhibition of NO synthesis reverses the profound vasodilation seen in *Cygb*^−*/*−^ mice with higher BP and SVR, normalization of CO, decreased vessel diameters and lower tissue perfusion, indicating that lack of Cygb greatly enhances NO-mediated vascular signalling.

### Effects of Cygb downregulation on Ang II-induced hypertension

It has been demonstrated that angiotensin II (Ang II)-induced hypertension is associated with enhanced superoxide generation in the vessel wall secondary to induction of vascular NADPH oxidase^41^,^42^,^43^. This increased superoxide is associated with vascular dysfunction due to superoxide-mediated NO consumption^41^. Since we observe that downregulation of Cygb in SMCs and vessels decreases vascular tone and BP with preservation of NO and potentiation of NO signalling, we hypothesized that a decrease in Cygb-mediated NO consumption may be able to compensate for the increase in superoxide-mediated NO consumption that occurs in the vessels of mice with Ang II-induced HTN. Therefore, we performed experiments to determine if this could be utilized to enhance endothelium-dependent vasodilation in order to ameliorate hypertension (HTN).

**Figure 7:**
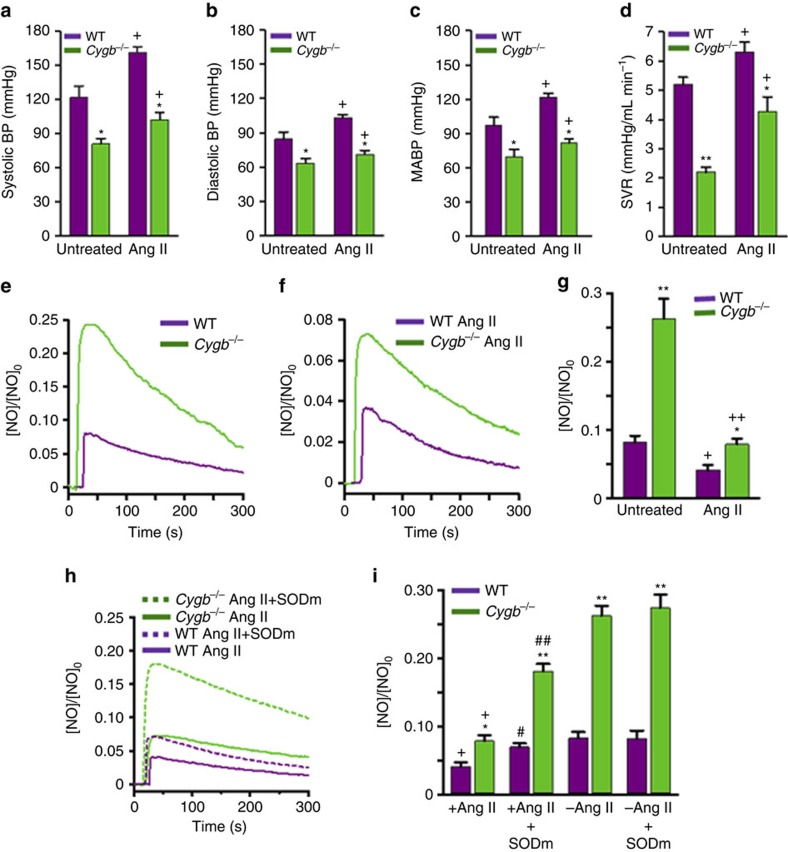
Effect of Ang II treatment on blood pressure, vascular resistance and NO metabolism. Systolic (**a**), diastolic (**b**), mean arterial blood pressure (MABP) (**c**) and systemic vascular resistance (SVR) (**d**) of mice with and without Ang II treatment. Measurements were performed after 4 weeks of Ang II administration by osmotic pump, *n*=5–7 per group. The flux of NO diffusion across the wall of mesenteric arteries from these control or Ang II-treated mice was measured by cylindrical carbon electrodes (**e**,**f**). NO was injected into the solution to achieve an initial concentration of 0.5 μM. The average NO diffusion flux through the wall of control or Ang II-treated mouse mesenteric resistance arteries, *n*=5 per group, is shown (**g**). Effect of SOD mimetic (SODm) on the flux of NO across the mesenteric artery wall of Ang II-treated WT and *Cygb*^−*/*−^ mice (**h**). Comparison of the flux of NO diffusion across the wall of mesenteric artery segments from untreated or Ang II-treated mice in the presence/absence of SOD mimetic (**i**), *n*=5 per group. *P<0.05, **P<0.01 WT versus *Cygb*^−*/*−^. ^+^*P*<0.05, ^++^*P*<0.01 WT or *Cygb*^−*/*−^ untreated versus Ang II treated. ^#^*P*<0.05, ^##^*P*<0.01 WT or *Cygb*^−*/*−^ Ang II treated versus Ang II treated+SODm. Error bars: mean±s.e.m.; *P* values determined using a two-tailed *t*-test.

Experiments were performed in a mouse model of Ang II-mediated HTN. WT and *Cygb*^−*/*−^ mice were chronically administered with Ang II by osmotic pump at doses known to induce HTN^44^,^45^,^46^. In WT mice, HTN was observed with systolic BP of 160 mmHg, diastolic BP of 104 mmHg and mean BP of 126 mmHg measured after 4 weeks of Ang II administration versus values of 120, 85 and 100 mmHg, respectively, in untreated WT mice (Fig. 7a–c). In contrast, *Cygb*^−*/*−^ mice did not develop HTN post-Ang II administration and BP values remained in the normal range with values of 105, 70 and 82 mmHg. Thus, downregulation of Cygb or its NOD function could provide a novel, highly potent approach to prevent or reverse HTN. In parallel with the lower BP values, SVR was also lower in the Ang II-treated *Cygb*^−*/*−^ mice compared to WT (Fig. 7d).

Measurements of the flux of NO diffusion across the wall of small resistance vessels from these control untreated WT and *Cygb*^−*/*−^ mice showed that peak NO flux across the mesenteric artery wall of *Cygb*^−*/*−^ mice was ∼3.2-fold higher than that of matched WT vessels. As expected, this difference, while large, is less than that measured in aorta which has a thicker wall (Fig. 5). Measurements of the flux of NO diffusion across the wall of mesenteric artery from Ang II-treated mice demonstrated that Ang II treatment decreased the measured NO levels with decreased NO flux due to increased rate of NO degradation in the wall of both WT and *Cygb*^−*/*−^ vessels (Fig. 7e–g). However, the NO flux in *Cygb*^−*/*−^ vessels remained much higher than in WT, with values similar to those in normal untreated WT vessels due to the lower rate of NO consumption in the wall of these vessels. Thus, the decreased rate of NO degradation with *Cygb* knockout lowered vascular resistance, preventing Ang II-induced hypertension.

Additional experiments were performed to assess the role of superoxide in the process of NO decay in the vessels of Ang II-treated mice using a SOD mimetic taken up in cells (GC4419, Galera Therapeutics, Inc.). In mesenteric arteries from *Cygb*^−*/*−^ mice, treatment with the SOD mimetic largely reversed the Ang II-associated decrease in NO diffusion flux with a 130% increase seen, while in WT vessels a 68% increase was seen, with values restored close to those in vessels not treated with Ang II (Fig. 7e,f,h,i). Thus, most of the increased NO consumption seen with Ang II treatment is confirmed to be secondary to the increased levels of superoxide induced in the vascular wall by Ang II. In WT and *Cygb*^−*/*−^ vessels, treatment with the SOD mimetic did not alter the NO diffusion flux, and curves were indistinguishable from those in Fig. 7e. Interestingly, statistical analysis shows that the effect of Cygb on NO flux and MABP depends on Ang II, with a highly statistically significant interaction between Cygb and Ang II. These results confirm that NO degradation with decreased NO diffusion flux through the wall of small resistance vessels can occur due to reaction with superoxide, as induced by Ang II, or due to dioxygenation by Cygb, and that both pathways of NO consumption interact, with each contributing to regulation of vascular tone and BP. We further observe that a decrease in Cygb expression or NOD activity could compensate for the increased superoxide-mediated NO consumption seen with Ang II administration and prevent hypertension.

### Smooth muscle versus endothelial NO consumption

**Figure 8:**
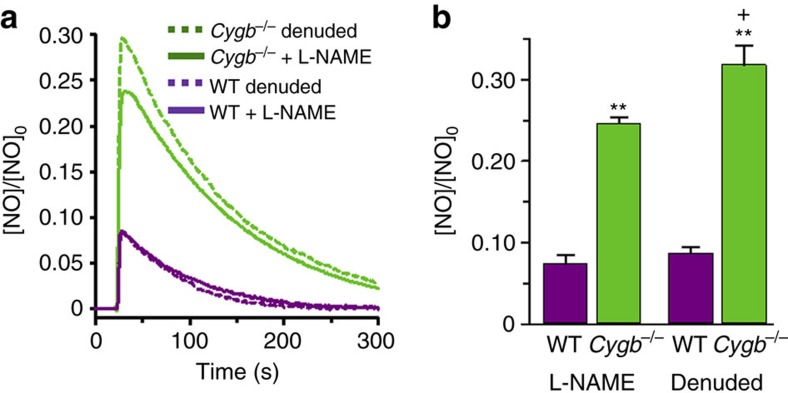
Effect of endothelium on the flux of NO diffusion across the wall of small resistance vessels isolated from WT and *Cygb*^−/−^ mice. The flux of NO diffusion across the wall of mesenteric artery (MA) segments with intact endothelium or denuded of endothelium (**a**). To prevent endogenous NO generation from eNOS, vessels with intact endothelium were treated with the NOS inhibitor L-NAME (1 mM). NO was injected into the solution to achieve an initial 0.5 μM concentration. Mean and standard errors of the measured NO flux across the wall of MA segments denuded of endothelium or with intact endothelium+L-NAME (**b**). Error bars: mean±s.e.m., *n*=5 per group. **P<0.01 WT versus *Cygb*^−*/*−^. ^+^*P*<0.05 *Cygb*^−*/*−^+L-NAME versus *Cygb*^−*/*−^ denuded; *P* values determined using a two-tailed *t*-test.

NO is primarily synthesized in the endothelium and then diffuses into the vascular smooth muscle, where it influences vessel tone. Recently, it has been reported that in small resistance vessels Hb-α is expressed in endothelial cells and enriched at the myoendothelial junction, where it can serve to regulate NO flux out of the endothelial cell to the vascular smooth muscle^14^,^30^. In order to assess the relative importance of endothelial versus smooth muscle mediated NO consumption, we performed additional experiments comparing measurements of NO flux across endothelium-denuded mesenteric artery vessels compared to vessels treated with L-NAME. As reported above, in *Cygb*^−*/*−^ vessels the NO flux was much higher than in WT (Fig. 8). The NO diffusion flux was observed to be almost identical in WT vessels with only a slight but not significant 6% higher flux in the endothelium-denuded vessels. Interestingly, in the *Cygb*^−*/*−^ vessels with much higher NO flux and much lower NO consumption, a small but significant 28% increase in NO flux was seen in the endothelium-denuded vessels. Thus, these results suggest that the major process of NO consumption that limits NO flux through the wall of small resistance vessels is the process of NO consumption by Cygb in the smooth muscle; however, there also appears to be a significant but smaller contribution lost in endothelium-denuded vessels consistent with the prior reports of a mechanism regulating NO flux at the myoendothelial junction^14^,^30^.

## Discussion

The function of Cygb has been debated since its first discovery just over a decade ago^31^,^34^. Roles in oxygen delivery, redox biology, cell signalling and NO regulation have been proposed^47^. Cygb is considered to have a common evolutionary ancestor with Mb^34^. In accordance with the literature^29^, we observe that Mb concentrations in vascular smooth muscle are very low, >40-fold lower than Cygb. This preferential expression of Cygb over Mb would suggest that there is an important functional benefit or role uniquely provided by Cygb. Indeed, we observed that Cygb has a uniquely fast reduction rate that is more than 10-fold to 100-fold faster than for Mb, depending on the reducing system, resulting in more than a 10-fold higher rate of NO consumption (Fig. 1). This higher rate of NO consumption by Cygb than Mb is consistent with prior reports^36^. In a similar manner, Hb-α expression was >200-fold less than Cygb and its reduction rate and rate of NO consumption was similar to Mb and more than 10-fold slower than Cygb. From this data, we can see that Cygb is a highly potent NOD that is efficiently and rapidly reduced by cellular reducing systems. Along with the relatively high expression level of Cygb in smooth muscle compared to that of other globins, this confers Cygb with a major role in the O_2_-dependent metabolism of NO. With knockdown of Cygb expression in SMCs from aorta or mesenteric artery, more than 70–75% of NO metabolism in vascular smooth muscle was shown to be Cygb dependent. Thus, Cygb serves as the major mechanism of NO degradation in vascular smooth muscle.

In addition to its high potency as an NOD, the NOD activity of Cygb has uniquely high O_2_ dependence. In general, the rate of NO decomposition by oxy-globins decreases when O_2_ concentrations decrease, as levels of nitrosyl globins increase and oxy-globins decrease as illustrated in Supplementary Fig. 1 and previously detailed^12^. With Cygb, hypoxia sharply decreases the rate of NO metabolism, while with Mb only a gradual linear decrease occurs^12^,^15^. It has been reported that in the O_2_ concentration range from 0 to 50 μM with ascorbate as reductant, the rate of NO dioxygenation by Cygb is over 100-times more sensitive to changes in O_2_ concentration than Mb^15^. Thus, Cygb is uniquely suited for O_2_-dependent regulation of NO levels and metabolism in vessels.

The decrease in the Cygb NOD function with physiological hypoxia has been hypothesized to preserve NO levels and tissue perfusion under hypoxia. Furthermore, under conditions of hypoxia, nitrite can be reduced back to NO serving as a NOS-independent pathway of NO generation^48^,^49^,^50^. With severe hypoxia progressing to anoxia, we have previously observed that reduced Cygb can reduce nitrite back to NO^8^,^11^, further enhancing NO and vasorelaxation under conditions of severe hypoxia. However, with the low P_50_ of O_2_ binding to Cygb of 1.5 Torr, Cygb-mediated NO production secondary to nitrite reduction is significant only at very low pO_2_, and would only be expected to be important during severe prolonged ischaemia, not in the normal physiological regulation of vascular tone. Thus, under normoxia, Cygb functions as an NOD, oxidizing NO to nitrate (Fig. 2) while under anoxic conditions, Cygb can reduce nitrite back to NO. Based on the unique O_2_ dependence of its effects on NO, Cygb has been proposed to have a role in O_2_-dependent flow regulation and hypoxic vasodilation^11^,^12^,^15^.

Under normal physiological conditions, endothelium-derived NO regulates vascular tone. NO is required for endothelium-dependent vasodilation which requires diffusion of eNOS-derived NO from the endothelium to the site of sGC in the vascular smooth muscle. Therefore, one might expect that the major NOD in vessels would be present in the smooth muscle where it would serve to regulate the magnitude and duration of sGC activation. Indeed, we observed that Cygb expression was present within the SMCs of the vessel wall and absent from the endothelium (Fig. 5c). With knockout of Cygb, the magnitude of endothelium-dependent or endothelium-independent vessel relaxation was greatly increased, with over a 20-fold shift in the vasodilation dose–response curves to acetylcholine or the NO donor SNP (Fig. 5). With knockout of Cygb, the diffusion flux of NO across the wall of the aorta was increased more than sixfold and across the much smaller mesenteric artery by more than threefold (Figs 5 and 7). Much higher cGMP levels were detected in freshly harvested, unstimulated vessels with fivefold higher levels than in WT vessels (Fig. 6d), confirming that Cygb expression regulates both NO degradation and sGC activation.

In addition to large effects on the function of *ex vivo* vessels, in *Cygb*^−*/*−^ mice, large alterations were seen on *in vivo* vascular tone, BP and cardiac function compared to the background matched WT mice. MABP and SVR values were markedly decreased by 30% and 54%, respectively (Fig. 6). Furthermore, CO was increased by 68% in the *Cygb*^−*/*−^ mice, likely as a compensation for the marked vasodilation present. Interestingly, NOS inhibition largely reversed the low MABP and SVR values as well as the elevated CO of the *Cygb*^−*/*−^ mice to values close to those in WT, confirming that these alterations were secondary to enhanced NOS-derived NO levels. From ultrasound measurements, the aorta was also observed to be dilated in *Cygb*^−*/*−^ mice compared to WT and this was also largely reversed by L-NAME. A marked increase in tissue perfusion was also observed in the *Cygb*^−*/*−^ mice compared to WT, further demonstrating vasodilation of the small resistance vessels that control tissue perfusion. This increased perfusion was also reversed by NOS inhibition. Together these results indicate that the NOD function of Cygb is of critical importance for the *in vivo* regulation of NO levels that in turn control vascular tone, BP, cardiac function and tissue perfusion.

In a wide variety of cardiovascular diseases, ranging from hypertension to atherosclerosis, impaired endothelium-mediated vasodilatory function occurs secondary to impaired NOS function or enhanced NO scavenging. Since we observed that downregulation of Cygb expression enhances endothelium-derived NO and secondary NO-mediated signalling and vasodilation, one can hypothesize that downregulation of Cygb-expression levels or NOD function could ameliorate or even serve to prevent cardiovascular disease. As Ang II-induced hypertension has been well demonstrated to be due to enhanced superoxide generation and secondary NO degradation^41^,^42^,^43^, we evaluated if downregulation of Cygb expression could prevent or ameliorate the onset of Ang II-induced hypertension. While, as expected, chronic Ang II administration induced hypertension in WT mice (Fig. 7a–c), in contrast, *Cygb*^−*/*−^ mice did not develop hypertension. In parallel with the lower BP values, SVR also remained lower in the Ang II-treated *Cygb*^−*/*−^ mice compared to WT (Fig. 7d). Measurements of the NO diffusion flux across the wall of resistance vessels from control or Ang II-treated mice demonstrated that Ang II treatment decreased NO flux due to an increased rate of NO degradation in the wall of both WT and *Cygb*^−*/*−^ vessels (Fig. 7e–g). This increased rate of NO degradation was secondary to enhanced superoxide generation, since it was largely reversed by a SOD mimetic (Fig. 7h,i). The NO flux in *Cygb*^−*/*−^ vessels remained much higher than in WT vessels, with values similar to those in normal untreated WT vessels, due to the lower rate of NO consumption in the wall of these vessels. Thus, downregulation of Cygb or its NOD function could provide a novel, highly potent approach to prevent or reverse hypertension.

From the current study, it is clear that NO degradation in the vascular wall is largely due to the NOD function of Cygb in the presence of cellular reducing systems. From siRNA-mediated knockdown experiments, b5R was shown to be the major reductase involved, with a 67% decrease in NO degradation rate estimated; however, other enzymatic or non-enzymatic reducing systems may also be involved, such as P450 reductase and ascorbate^12^,^15^,^36^,^51^. Interestingly, b5R has also been reported to be of critical importance for the process of Hb-α mediated NO dioxygenation at the myoendothelial junction that has been reported to regulate NO flux out of the endothelial cell to the vascular smooth muscle of small resistance vessels^14^,^30^. In an effort to assess the role of endothelial factors such as Hb-α on the overall process of NO metabolism in small resistance vessels, we measured NO flux across endothelium-denuded mesenteric artery vessels compared to vessels treated with the NOS inhibitor L-NAME. In *Cygb*^−*/*−^ vessels, the NO flux was much higher than in WT vessels (Fig. 8). While the NO diffusion flux in WT vessels showed only a slightly but not significantly higher flux in the endothelium-denuded vessels, in the *Cygb*^−*/*−^ vessels, with much higher NO flux and lower NO consumption, a significant 28% increase in NO flux was seen in the endothelium-denuded vessels. Thus, the major process of NO consumption that limits NO flux through the wall of small resistance vessels is due to NO consumption by Cygb in the smooth muscle; however, there also appears to be a significant but smaller contribution from the endothelium, consistent with the prior reports of a mechanism regulating NO flux at the myoendothelial junction^14^,^30^.

In conclusion, we demonstrate that Cygb has a critical role in regulating *in vivo* vascular tone, BP and cardiovascular function. Cygb is shown to be the main pathway of NO metabolism in vascular smooth muscle, regulating NO flux through resistance and conduit vessels. Downregulation of Cygb ameliorated Ang II-mediated hypertension. Since impaired endothelium-dependent NO signalling is a central trigger of a wide range of cardiovascular disease (from hypertensive, to diabetic, to atherosclerotic), downregulation of Cygb or its NOD function, in order to enhance vascular NO levels and restore protective NO signalling, could provide a much needed remedy. Therefore, therapeutic approaches to modulate Cygb expression and its NOD function could be of great value in the prevention and amelioration of cardiovascular disease.

## Methods

### Knockdown of Cygb or b5R in aortic and mesenteric SMCs

Rat aSMCs (Lonza Walkersville, Inc., Walkersville, MD) or mesenteric arterial SMCs were prepared, characterized and cultured in our laboratory according to previous studies^52^,^53^,^54^,^55^,^56^,^57^. Briefly, under aseptic conditions, the vessel was carefully dissected out from the rat, cleaned of extraneous tissues under the dissecting microscope, placed in a sterile HBSS and washed. The vessel was transferred to a 35 mm culture dish containing 347 U ml^−1^ collagenase solution (type 2, 347 U mg^−1^; Worthington Biochemical) and incubated at 37 °C for ∼45 min. The vessel was transferred into a dissection dish containing HBSS and the ends were gently pinned down then cut open longitudinally, with the luminal surface upward. The endothelium was removed by scraping the cell layer off under the dissecting microscope. The vessel (without adventitia) was gently transferred to a 35 mm culture dish containing pre-warmed culture medium and incubated overnight. The vessel was again gently transferred to a 35 mm culture dish containing 347 U ml^−1^ collagenase solution and 3 U ml^−1^ elastase solution (type IV, 6 U mg^−1^; Sigma-Aldrich) and incubated at 37 °C for ∼30 min. The partially digested tissue in the enzyme solution was transferred to a 15 ml conical tube and carefully tritrated, with a sterile Pasteur pipette (1–1.5 mm tip opening) with attached rubber bulb, until the tissue dissolved and the cells dissociated. A 10 ml aliquot of cultured medium was added to stop the enzyme digestion. A 10 μl sample of suspension was tested for the appearance of single cells. The cell suspension was centrifuged at 1,500 r.p.m. for 5 min. The supernatant was carefully aspirated and 2–5 ml of pre-warmed complete culture medium was added to re-suspend the cells at a density of about 8 × 10^5^ cells per ml. Cells were then seeded into 25 cm culture flasks and placed into a 37 °C, 5% CO_2_ incubator. Within 24 h, all viable cells were attached and the medium was then replaced with fresh pre-warmed complete culture medium. Half of the culture medium was replaced every 2–3 days until a confluent SMC monolayer was obtained.

Cells were characterized morphologically (hill and valley, elongated and spindle-like shape) as well as by checking the expression of myosin heavy chain (in freshly prepared cells) and smooth muscle-specific α-actin, the most specific smooth muscle markers. Cells were cultured and passaged in DMEM:F12 supplemented with 20% heat-inactivated fetal bovine serum, GA-1000 and 50 IU ml^−1^ penicillin/streptomycin (50 IU ml^−1^/50 mg ml^−1^) according to the Lonza protocol. Experiments were performed using 70–80% confluent cells at passages 4–6.

SMCs were transfected with Cygb siRNA or cytochrome b5 reductase 3 (b5R) siRNA (Santa Cruz Biotechnology) using Lipofectamine RNAiMAX (Invitrogen) according to the manufacturer's recommendations^11^. Briefly, Lipofectamine RNAiMAX was mixed gently by pipetting up and down with antibiotic-free Opti-MEM medium (6 μl:100 μl ratio for each 1 ml growth medium) and incubated at room temperature for 5 min. An aliquot of Cygb or b5R siRNA (final concentration of 100 nM) was mixed with the Lipofectamine RNAiMAX/Opti-MEM mixture and incubated at room temperature for 30 min. In total, 1 ml of each of the mixtures was added to a separate 15 cm culture well containing SMCs exponentially growing in 9 ml of antibiotic-free Opti-MEM medium. Cygb, b5R and the corresponding scrambled siRNA-transfected cells were incubated at 37 °C in a 5% CO_2_-humidified incubator for 7 h, and Opti-MEM medium was changed to DMEM:F12 complete medium to provide essential nutrients and growth factors for optimal growth and cell survival. Forty-eight hours post transfection, Cygb, b5R or the corresponding scrambled siRNA-transfected cells were collected for further studies and protein expression was evaluated by Western blotting.

### SDS–polyacrylamide gel electrophoresis and immunoblotting

Whole-cell lysates or pure proteins, in RIPA buffer, were quantitated using a Bio-Rad DC protein assay kit. The standard procedures for SDS–polyacrylamide gel electrophoresis and immunoblotting were followed as described previously^58^. Proteins were separated at room temperature on a reducing graded (4–20%) Tris-glycine polyacrylamide gel at 125 V. Protein bands were transferred electrophoretically to a PVDF membrane in 12 mM Tris-HCl, 96 mM glycine, 20% methanol using an Xcell II Blot Module (Invitrogen) at 25 V constant for 90 min. The following antibodies (Santa Cruz Biotechnology) were used: rabbit polyclonal anti-Cygb (sc-66855; diluted 1:200), anti-Mb (sc-25607; diluted 1:200), anti-Hb-α (sc-21005; diluted 1:200), cytochrome b5 reductase 3 (sc-398043; diluted 1:200) and mouse monoclonal anti-actin (sc-47778; diluted 1:500). HRP linked anti-mouse and anti-rabbit (Cell Signaling Technology; 7,076 and 7,074, respectively) were used as secondary antibodies at a dilution of 1:3,000. Membranes were blocked for 1 h at room temperature in Tris-buffered saline (TBS) containing 0.05% Tween 20 (TBST), with 5% dried milk and incubated overnight with primary antibodies at 4 °C. Membranes were then washed three times in TBST, incubated for 1 h with horseradish peroxidase-conjugated secondary antibody in TBST at room temperature and again washed three times in TBST. Protein bands were then detected with ECL Western Blotting detection reagents (Amersham Biosciences) and exposed to an X-ray film. Protein band densities were quantified by a high resolution Pharos FX Plus Molecular Imager (Bio-Rad). The protein concentration was obtained by the quantitation of the band densities using a high resolution Pharos FX Plus Molecular Imager (Bio-Rad) of Cygb and Mb and comparing them to band densities of pure protein standards run in parallel^11^.

### Measurement of ferric globin reduction

The reduction of globins by a reductant (Asc) or a reducing system (cytochrome b5 reductase (b5R)/b5/NADH) was performed in a cuvette under anaerobic conditions. The reaction was monitored using a Cary 50 ultraviolet/vis spectrophotometer by measuring the changes in absorbance at 416 nm for Cygb(Fe^3+^), 410 nm for Mb(Fe^3+^) or 406 nm for Hb-α(Fe^3+^) with time at 37 °C^51^. After addition of 1.5 ml buffer solution and placement of the Clark O_2_ electrode, the cuvette was sealed with a parafilm membrane. An Apollo 4,000 Free Radical Analyzer (WPI Inc., Florida) was used along with the Clark electrode to monitor O_2_. The solution was stirred using a magnetic stir bar at the bottom of the cuvette. An argon gas tube was inserted in the cuvette to bubble argon into the solution for 15 min to quickly remove O_2_. Before injecting a sample of ferric globin into the test solution, the argon gas tube was removed from the solution and placed above the solution surface to keep an argon flow in the cuvette. About 20 min after injecting a sample of ferric globin (3 μM) for reduction by Asc or ferric globin (3 μM)+b5 (0.5 μM)+NADH (100 μM) for reduction by b5R/b5/NADH into the solution, either Asc (10 mM) or b5R (30 nM) was added to initiate reduction of the ferric globin. From the recorded kinetic curves, the initial rates of Cygb(Fe^3+^), Mb(Fe^3+^) or Hb-α(Fe^3+^) reduction and their rate constants were determined.

### Immunofluorescence of expression of Cygb and eNOS in the mouse aorta

Mouse aorta was isolated and placed in a block holder containing OCT embedding compound and snap frozen in dry ice. Sections from the blocks were cut at 4 μm on a cryotome and processed for immunostaining. The frozen sections were then blocked with 1% BSA in TBST, incubated with primary rabbit polyclonal anti-Cygb and mouse anti-eNOS antibodies (Santa Cruz Biotechnology) in TBST (1:500 dilutions)+1% BSA for 1 h at room temperature, followed with the incubation of respective secondary goat anti-mouse Alexa Fluor 488-conjugated and goat anti-rabbit Alexa Fluor 568 (1:1,000 dilutions) as necessary, for 1 h at room temperature. After washing with TBS-T, the sections were mounted in anti-fade mounting medium (Fluoromount-G, Birmingham, AB) and examined using an Olympus FV 1,000 confocal microscope (Olympus America Inc., Melville, NY) with the × 40 objective with 405, 488 and 568 nm excitations for DAPI, green and red fluorescence, respectively^59^.

### Preparation of NO stock solutions

NO stock solutions for these experiments and the work that follows were prepared as described previously^35^,^60^. The preparation process was performed in a fume hood. Briefly, NO gas was scrubbed of higher nitrogen oxides by passage first through a U-shaped tube containing NaOH pellets and then through a 1 M deaerated (bubbled with 100% argon) KOH solution, in a custom-designed apparatus using only glass and/or stainless steel tubing and fittings (no plastic components). The purified NO was collected by saturating a deaerated phosphate buffer solution (0.2 M potassium phosphate, pH 7.4) contained in a glass sampling flask (Kimble/Kontes, Vineland, NJ) fitted with a septum for anaerobic extraction of the NO solution with a gas-tight Hamilton syringe (Hamilton Robotics, Reno, NV).

### Measurements of the rate of NO metabolism by SMCs

Measurements were performed in a four-port water-jacketed electrochemical chamber (NOCHM-4, WPI, Sarasota, FL) at 37 °C in air-equilibrated buffer as described in our previous papers^15^,^61^. The solution was rapidly stirred with a magnetic bar during the NO measurements. Two Clark-type NO electrodes (NOCHM-4, WPI, Sarasota, FL) were placed in the chamber through two ports on the side wall of the chamber for measuring the rate of NO metabolism by isolated normal SMCs, Cygb knockdown (Cygb siRNA) SMCs or cytochrome b5 reductase 3 knockdown (b5R siRNA) SMCs. Before adding the SMCs into the solution, we first measured the rate of NO decay in the buffer solution. Then, 7 × 10^6^ per ml SMCs were added into the chamber and the rate of NO decay was measured after NO was injected into the solution to achieve an initial concentration of 0.5 μM using a 10 μl gas-tight Hamilton syringe.

### Construction of *Cygb* ^−/−^ mice

C57BL/6 Cygb knockout (*Cygb*^−*/*−^) mice were used derived from the colonies previously generated as described^62^. Briefly, mice lacking exon 1 of the Cygb gene were generated using the lox-P system^63^. The targeting vector (pTVneo/Cygb) was constructed from PCR DNA fragments from SV129 mouse genomic DNA. Embryonic stem cells (1 × 10^7^ cells per ml) were transfected with a linearized targeting vector (20 μg) by electroporation and cultured in selection medium containing 150 μg ml^−1^ geneticine (G418). Homologous recombinant clones were aggregated with C57BL/6-DBA2 F1 mouse morulae and one produced chimeric mice that transmitted the knockout construct. Chimeric males were mated with C57BL/6J females to obtain Cygb heterozygous mice which were backcrossed to the C57BL/6J background for more than nine generations (mice purchased from Japan SLC, Inc., Tokyo, Japan). Cygb heterozygous mice were intercrossed. The litter sizes were normal and analysis of the tail biopsies at 4 weeks of age from 102 offspring from heterozygote crosses revealed the presence of homozygous mutant mice at a frequency of 24%. The homozygotes appeared normal morphologically and histopathologically 1 month after birth. All mice were cared for according to the guidelines approved by the Institutional Animal Care and Use Committees of Osaka City University and The Ohio State University.

### Measurements of NO diffusion and NO metabolism in the aortic wall

The flux of NO diffusion across the aortic wall was measured by Clark-type NO electrodes^35^. Aortas were excised from adult, male *Cygb*^−*/*−^ mice or age-matched adult, male C57BL/6J mice of 9–12 months of age. A segment of aortic ring was longitudinally opened and the opened aortic wall was placed on an NO electrode with an aorta attachment as depicted in Supplementary Fig. 2. The flux of NO diffusion across the aortic wall was recorded by the aortic-wall-covered electrode after NO was injected to provide an initial concentration of 3 μM in the surrounding solution. [NO] in the solution was recorded by a second NO electrode.

### Mesenteric artery dissection and cannulation by electrode

Adult, male *Cygb*^−*/*−^ mice or age-matched adult, male C57BL/6J mice (9–12 months of age) were anaesthetized using an intraperitoneal injection of ketamine (100 mg kg^−1^) and xylazine (10 mg kg^−1^). First and second order mesenteric arteries (1st and 2nd branch from superior mesenteric artery) were dissected in physiological saline solution bubbled with 95% O_2_/5% CO_2_ and isolated from surrounding adipose and connective tissue using a dissecting microscope and mounted onto the tip of a micro-cylindrical carbon electrode for measurements of NO diffusion kinetics as depicted in Supplementary Fig. 3. For denuded endothelium experiments, vessels were mounted onto the tip of the carbon electrode as described above, but were gently rotated around the electrode three times before experiments. The flux of NO diffusion across the mesenteric arterial wall was recorded by the carbon electrode after NO was injected to provide an initial concentration of 0.5 μM NO in the solution. [NO] in the solution was recorded by a second Clark-type NO electrode or carbon NO electrode. To prevent the effect of endogenous NO generation from eNOS in the vascular wall, 1 mM L-NAME (a NOS inhibitor) was added to the solution in experiments using vessels with intact endothelium. To examine the effect of Ang II-induced vascular superoxide (O_2_^·−^) on NO diffusion in the wall of mesenteric resistance arteries with Ang II treatment, the flux of NO across the arterial wall was measured as described in the absence and presence of 50 μM superoxide dismutase mimetic (SODm) (GC4419, Galera Therapeutics, Inc.).

### Mouse aortic ring preparation for vascular function measurements

Preparation of the isolated mouse aorta was similar to that previously described^64^. Briefly, the thoracic aorta was gently dissected from anaesthetized and heparinized adult, male *Cygb*^−*/*−^ mice or age-matched adult, male C57BL/6J mice (9–12 months of age), carefully cleaned of fat and connective tissues, and cut transversely into rings of 2–3 mm in length. The rings were mounted on a wire myograph (Multi Myograph System-610M, Danish Myo, Aarhus, Denmark) with care taken not to damage the endothelium, and then suspended in 5-ml organ baths containing modified KHB (containing (in mM) 118 NaCl, 24 NaHCO_3_, 4.6 KCl, 1.2 NaH_2_PO_4_, 1.2 CaCl_2_, 4.6 HEPES, and 18 glucose) and continuously purged with 95% O_2_–5% CO_2_ (37 °C, pH 7.4). Aortic rings were equilibrated for 90 min with an initial resting tension of 1 g, and the bathing solution was changed at 15-min intervals. Changes in isometric tension were recorded on a PowerLab/8sp multichannel data-acquisition system (AD Instruments, Colorado Springs, CO) using ADI Chart software (version 5.3) for digital processing and data analysis. After equilibration, the responsiveness and stability of each ring was checked by the successive administration of a maximally effective concentration of L-phenylephrine hydrochloride (phenylephrine; 1 μM). The integrity of the vascular endothelium was assessed pharmacologically by acetylcholine (Ach)-induced relaxation of phenylephrine-pre-contracted rings. Preparations were then washed three times with drug-free buffer and allowed to relax fully for 30 min before the experimental protocol began. To determine the vasodilatory response to ACh, the aortic rings were pre-contracted with phenylephrine, and dose–response curves for aortic relaxation were obtained by the cumulative addition of Ach or sodium nitroprusside to the organ bath. The concentration of agonist in the organ bath was increased in steps of 1-log units. ACh was added to yield the next higher concentration only when the response to the lower dose reached a steady state. One dose–response curve for ACh was constructed for each ring. The vasodilator (relaxant) responses were expressed as per cent decreases of phenylephrine-induced pre-contraction, where the contraction produced by 1 μM phenylephrine in each ring from its initial resting tension (1 g) was considered as 100%.

### Blood pressure, CO and SVR measurements

Blood pressure was measured by non-invasive tail-cuff method in conscious adult, male *Cygb*^−*/*−^ mice or age-matched adult, male C57BL/6J mice (9–12 months of age) using a CODA high-throughput acquisition system (Kent Scientific, Torrington, CT). Briefly, mice were placed on a warming platform and allowed to acclimatize for 10 min before readings were obtained. Mice were trained for 7 days by measuring BP daily, after which BP recordings were made twice a week. Each session consisted of five acclimatization cycles followed by 15 BP measurements cycles. On the data collection day, two sessions of 15 BP measurements were obtained and the average of accepted readings from both sessions was used for systolic, diastolic, and mean BP in each individual mouse^65^. The computer software of the CODA system measures the systolic and diastolic pressures with inflation of a pneumatic tail cuff with a transducer that measures the BP waveform. If the mouse moves during measurement the noise distorts the waveform and the system software cannot measure the BP. Thus the software determines signal to noise and accepted readings, discarding noisy inaccurate measurements. Cardiac output was calculated from heart rate (HR) and stroke volume (SV) measured with a VisualSonics VEVO 2,100 Ultrasound System using the equation CO=HR × SV. Systemic vascular resistance (SVR) was calculated from the following: SVR=MABP/CO. For L-NAME experiments, L-NAME was dosed intraperitoneally at 300 mg kg^−1^ per day in PBS for at least 6 days.

### Measurement of cGMP levels in mouse aorta

Adult, male *Cygb*^−*/*−^ mice or age-matched male C57BL/6J mice (6–9 months of age) were anaesthetized by intraperitoneal injection of 100 mg kg^−1^ ketamine and 10 mg kg^−1^ xylazine. Descending aortas were dissected from the mice and quickly cleaned of adhering fat and connective tissue under a dissecting microscope in PBS. Cleaned aortas were homogenized in 0.1 N HCl and centrifuged at 10,000*g*. Supernatant was collected and used for ELISA detection of cGMP (Enzo Life Sciences, Farmingdale, NY). The acetylated format of the assay was used in order to improve sensitivity. Acetylation was performed by 1:20 addition of the acetylating reagent (1:2 acetyl anhydride:triethylamine) to the samples, which were then subjected to the ELISA assay according to the manufacturer's instructions. Aortic cGMP levels were expressed as picomoles per mg of protein in homogenates.

### Echocardiography

Transthoracic echocardiography was performed using the VisualSonics Vevo 2100 system. Adult, male *Cygb*^−*/*−^ mice or age-matched adult, male C57BL/6J mice (9–12 months of age) were anaesthetized using 2.0% isoflurane in 95% O_2_/5% CO_2_ at a rate of ∼0.8 l min^−1^. Anaesthesia was maintained by administration of oxygen and ∼1% isoflurane. Electrode gel was placed on the ECG sensors of the heated platform and the mouse was placed supine on the platform to monitor electrical activity of the heart. A temperature probe was inserted into the rectum of the mouse to monitor core temperature. The MS-400 transducer was used to collect the contractile parameters of the heart in the short axis M-mode. The aortic diameter of the heart was measured from the long axis B-mode.

### Angiotensin-II delivery and osmotic pump insertion

Adult, male *Cygb*^−*/*−^ mice or age-matched adult, male C57BL/6J mice (8–10 months of age) were anaesthetized with isoflurane. Under sterile conditions, a dorsal midline incision was made and a subcutaneous pocket was created in the right flank area. Alzet mini-osmotic pumps (Model 2004) (Durect Corp., Cupertino, CA) loaded with 200 μl saline or angiotensin-II (Ang-II) 7.2 mg ml^−1^ (Sigma-Aldrich, St. Louis, MO) were inserted subcutaneously to deliver Ang-II at 1 μg kg^−1^ min^−1^ for a period of 4 weeks^66^.

### Perfusion imaging

Perfusion imaging was performed using a Perimed laser speckle imager (Perimed Inc., Stockholm, Sweden). Perimed PimSoft software was used for acquisition and processing the data. Images were taken at a scan rate of 100 images/sec with a field of view of 24 cm × 24 cm. The CCD camera resolution was 1,388 × 1,038 pixels with a magnification up to 20 μm per pixel. Focusing and adjusting of field of view were done using a square low power laser light and a measurement distance of 10 cm was maintained during all acquisitions. Perfusion data were collected for 20 s with image resolution of 0.1 mm. The images were acquired at resting heart rate condition on the hairless, ventral side of 1% isoflurane-anaesthetized adult, male *Cygb*^−*/*−^ mice or age-matched adult, male C57BL/6J mice (9–12 months of age). For L-NAME experiments, L-NAME was dosed intraperitoneally at 100 mg kg^−1^ in PBS 12 h and 1 h prior to experiments.

### NOx measurements

Nitrite and nitrate were measured using an ENO-20 NOx analyzer (EiCOM Corp., San Diego CA). Each 100 μl sample consisted of 2 μM recombinant human Cygb, 150 U ml^−1^ Mn-SOD, 2.5 mM ascorbate and 50 μM DEA-NONOate (1,1-diethyl-2-hydroxy-2-nitroso-hydrazine sodium) in PBS with 0.1 mM EDTA, pH 7.4. After mixing, each sample was placed in an incubator shaker at 37 °C, 150 r.p.m. for 60 min. Injection volume into the ENO-20 instrument was 10 μl. The peak areas were determined using the eDAQ PowerChrom software provided with the instrument. Conversion to molarity was done by calibration against a range of concentrations of samples prepared from sodium nitrite and sodium nitrate run on the ENO-20 just prior to the experimental samples. Controls included all of the sample components except the Cygb (*n*=4 for both the Cygb and control).

### Protein purification

Recombinant human cytoglobin was purified as previously reported with some modifications^15^. The expression plasmid for Cygb (human Cygb cDNA in pET3ac, Novagen, Merck KGaA, Darmstadt, Germany) was obtained from Thorsten Burmester (Institute of Zoology and Zoological Museum, University of Hamburg, Germany) and transformed into *Escherichia coli* strain C41(DE3)pLysS. Cells were grown overnight in a 4 l flask in an incubator shaker at 37 °C in 1 l of Terrific Broth (47.6 g l^−1^) supplemented with glycerine (8 ml l^−1^), ampicillin (0.2 g l^−1^) and chloramphenicol (0.05 g l^−1^). The following morning, the cells were induced with IPTG (0.24 g l^−1^), the flask was sealed with parafilm and the bacteria was grown for an additional 6 h at 30 °C with the shaker set to 100 r.p.m. (decreased from 180 r.p.m.). The cells were harvested by centrifugation (3,000 r.p.m. for 30 min) and the cell pellet was resolubilized in 100 ml of 50 mM Tris-HCl pH 7.5, 0.5 M NaCl, 1 mM EDTA, 2 mM dithiothreitol, a pinch of lysozyme and deoxyribonuclease I, and Roche Complete Protease Inhibitor tablets (as recommended by manufacturer). The cells were placed in a stainless steel 250 ml beaker immersed in ice and lysed by sonication with a Branson Digital Sonifier equipped with a 1/2″ horn, using four 2 min repetitions with 10 min cooldown steps between each repetition. Insoluble matter was removed by centrifugation at 45,000*g* for 1 h in a high-speed centrifuge. A 35% ammonium sulfate precipitation was performed on the supernatant, the pellet was discarded, and the supernatant was dialyzed against 2 l of 50 mM Tris/HCl, 1 mM dithiothreitol and 0.1 mM EDTA, pH 7.5, with a total of three buffer exchanges. After dialysis, insoluble material was removed by centrifugation (45,000*g* for 1 h), and the protein was concentrated to 50 ml using Amicon Ultra-15 centrifugal filters (Millipore) with a 10,000 molecular weight cut-off. Further purification was performed with a GE Healthcare AKTA Purifier system with a 50 ml Superloop (GE Healthcare, Piscataway, NJ, USA) for sample loading. A HiPrep 16/10 DEAE FF anion-exchange column (GE Healthcare) was run with sodium chloride gradient elution, followed by a HiPrep 26/60 Sephacryl S-300 high-resolution size-exclusion column (GE Healthcare) eluted with 50 mM Tris/HCl, pH 7.5, 100 mM NaCl and 0.1 mM EDTA. The protein was concentrated and stored in 50 μl aliquots at −80 °C.

Haemoglobin α chains were purified as previously described with minor modifications^67^,^68^. 0.5 g of lyophilized human haemoglobin (Sigma) was solubilized in 2 ml of 0.25 M NaCl in H_2_O, pH 6–6.5. This solution was centrifuged at 45,000*g* to remove debris, oxidized with a crystal of solid potassium ferricyanide, and run down a 2 cm × 35 cm column of Sephadex G-25 equilibrated in the NaCl solution. The haemoglobin was concentrated with Amicon spin filters (30 kDa MWCO, Millipore) to 1 ml volume, made anaerobic by blowing a stream of argon gas over the solution for 30 min in a 15 ml conical tube, reduced with solid dithionite, then again run through the G-25 column to generate HbO_2_. The procedure of Yonetani and colleagues was then followed to purify the haemoglobin α chains using a 10-fold excess of 4-(hydroxymercuri)benzoic acid (HMB) to Hb tetramer as described^67^. The HMB was allowed to react with the HbO_2_ overnight at 4 °C, and the next morning the solution was centrifuged at 45,000*g* for 30 min to remove precipitate. The supernatant was run down a 2 cm × 35 cm Sephadex G-25 (fine, GE Healthcare) column to remove excess HMB, and the eluent was then loaded on a 3 cm × 10 cm DEAE-Sepharose (Sigma) column equilibrated in 10 mM potassium phosphate buffer, pH 8.0. The Hb-α eluted during the column wash step, and the fractions were pooled and concentrated using Amicon spin filters (10 kDa MWCO, Millipore) for removal of the HMB. Dithiothreitol (4 mM final concentration) was added to the concentrated protein sample and immediately applied to a 2 cm × 35 cm Sephadex G-25 column equilibrated in 100 mM Tris-HCl, 100 mM NaCl and 1 mM EDTA, pH 8.5 to remove bound HMB^68^. The eluent protein was concentrated (Amicon spin filters, 10 kDa MWCO, Millipore), aliquoted and stored at −80 °C until used.

### Data availability

The data that support the findings of this study are presented in the manuscript and the accompanying Supplementary Information file and can be obtained from the corresponding authors upon request.

## Additional information

**How to cite this article:** Liu, X. *et al*. Cytoglobin regulates blood pressure and vascular tone through nitric oxide metabolism in the vascular wall. *Nat. Commun.* **8,** 14807 doi: 10.1038/ncomms14807 (2017).

**Publisher's note**: Springer Nature remains neutral with regard to jurisdictional claims in published maps and institutional affiliations.

## Supplementary Material

Supplementary Information Supplementary Figures and Supplementary References
