# Supplementary material for: Cytoglobin regulates blood pressure and vascular tone through nitric oxide metabolism in the vascular wall
Source: Nat Commun. 2017 Apr 10;8:14807. doi: 10.1038/ncomms14807 (PMC5394235; doi:10.1038/ncomms14807)
Supplement: Supplementary Information — Supplementary Figures and Supplementary References [file ncomms14807-s1.pdf]

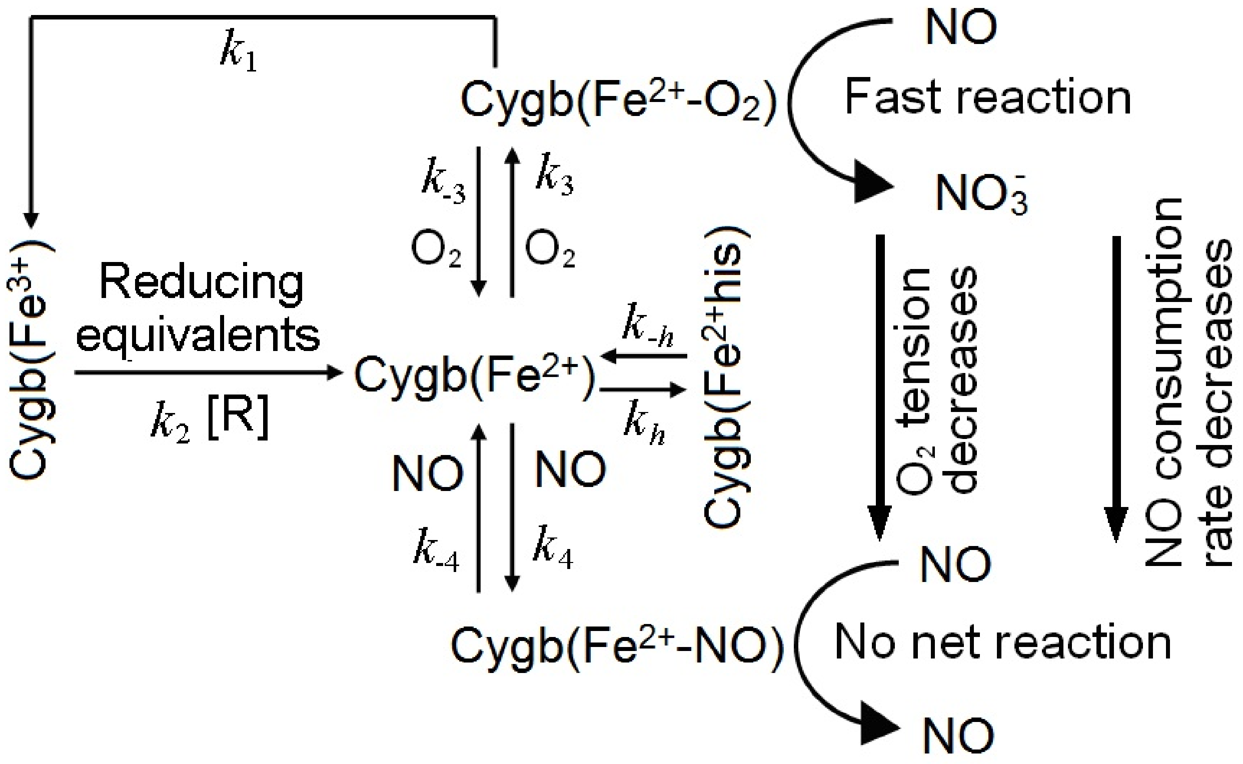

Supplementary Figure 1. Reaction mechanism of Cygb-mediated NO consumption. Based on prior *in vitro* studies, a reaction scheme for the molecular mechanism of O_2-regulated NO consumption by isolated Cygb has been developed as delineated above. By using a steady-state approximation approach, we derive the rate equation for NO consumption by Cygb in the presence of a cellular reductant [1], which can be further approximated by the equation:

$$V_{NO} \approx \frac{k_r[E][O_2]}{k_r(a+b[NO])+[O_2]} = \frac{V_{\max}[O_2]}{k_r(a+b[NO])+[O_2]}$$

where [*E*] is the total Cygb concentration or a sum of different forms of Cygb species (Cygb(Fe^{2+}), Cygb(Fe^{2+}-NO), Cygb(Fe^{2+}-O_2), Cygb(Fe^{3+}))

$$k_r = k_2[R], \quad V_{\max} = k_r[E], \quad a = \frac{k_{-3}k_4}{k_3k_{-4}k_1}, \; b = \frac{k_4}{k_3k_{-4}}$$

where [*R*] is the concentration of reductant. When this is applied to cells, the parameters k_r and [*E*] are related to cellular reductants and the total intracellular Cygb concentrations, so k_r and [*E*] may vary with cell type.

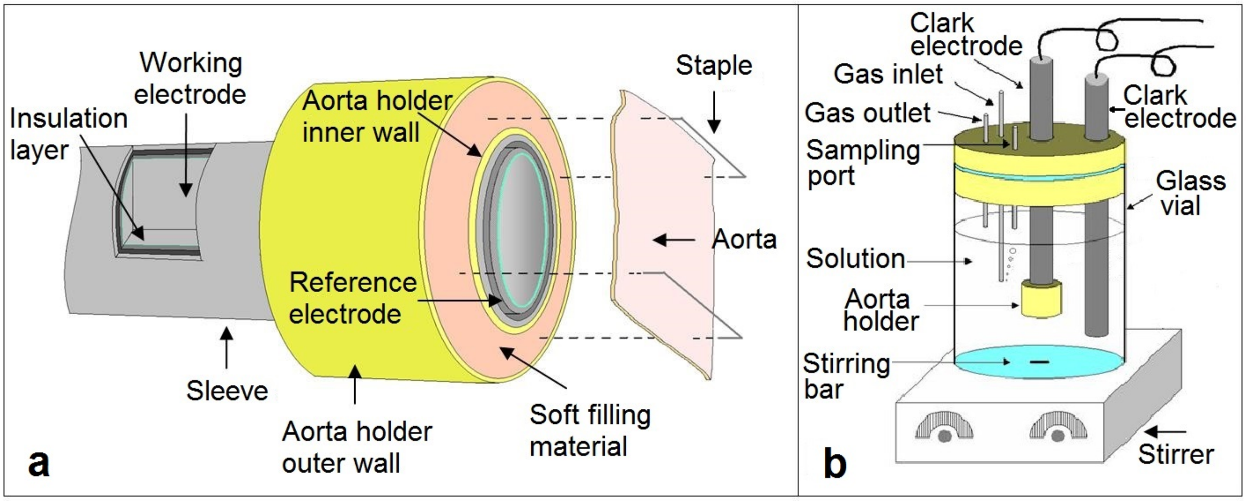

Supplementary Figure 2. Experimental setup for measuring NO diffusion in the aortic wall. The experimental setup for measuring the flux of NO diffusion across a segment of aorta (**a**) and the Clark NO electrodes with/without the aortic wall (**b**). The Clark electrode as shown in (**a)** consists of two integrated electrodes in a concentric structure with a central working electrode and an outer reference electrode separated by an insulation layer. Two Clark electrodes are inserted into the chamber containing tissue buffer solution through a cap: one is used to monitor NO concentration in the solution; the second is covered with a segment of aorta for measuring the flux of NO diffusion across the vascular wall. The chamber is temperature-controlled. Three additional ports in the cap are used for bubbling argon gas and/or injecting NO into the solution. The solution is stirred with a magnetic stirring bar.

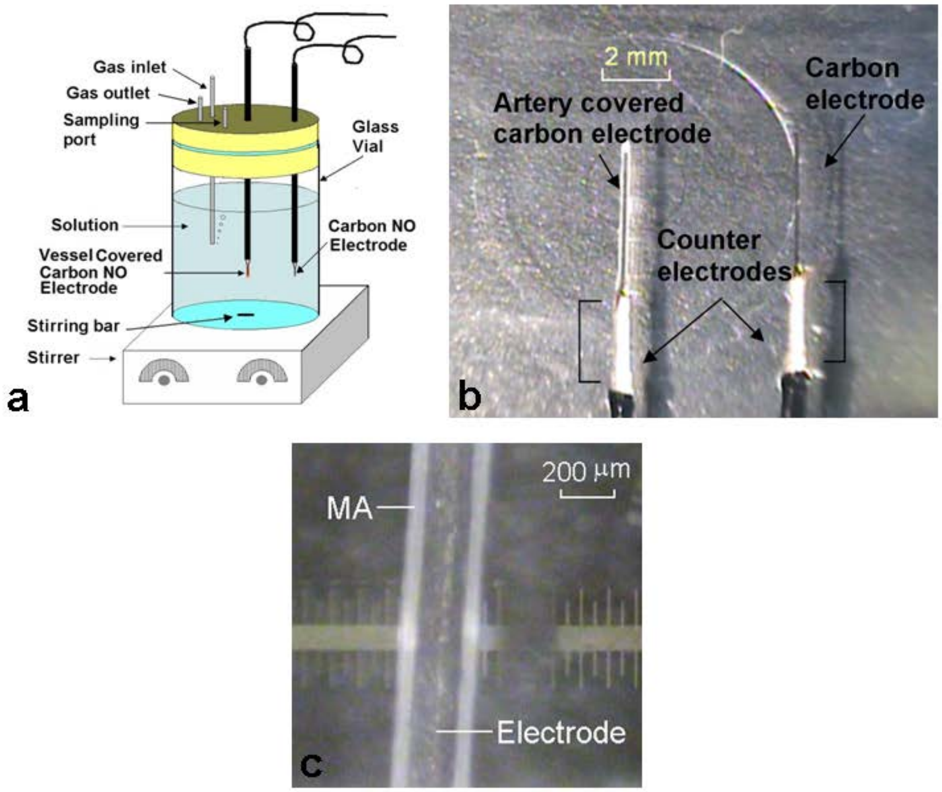

Supplementary Figure 3. Experimental setup for measuring NO diffusion in the mesenteric artery (MA) wall. The full setup for measuring the flux of NO across a segment of mesenteric resistance vessel (**a**) and the carbon NO probes with/without the resistance vessel (**b**). Two carbon microelectrodes are inserted into the chamber containing tissue buffer solution through a cap: one of them is used to monitor NO concentration in the solution; the second is covered with a MA for measuring the flux of NO diffusion across the vascular wall. The chamber is temperature-controlled. Three additional ports in the cap are used for bubbling argon gas and/or injecting NO into the solution. The solution is stirred with a magnetic stir bar. The tips of the two carbon composition electrodes are shown in **b**. The left one is covered by a MA segment. The black portion in each tip is the carbon NO electrode. To measure NO diffusion across the wall of MAs, the endothelium is removed by gently rubbing the intimal surface [2,3], and then a cylindrical carbon electrode is inserted into the lumen of the cleaned MA at one end and is slid toward the other end until the whole carbon electrode is covered completely by the MA. Enlarged image of the cylindrical carbon electrode covered by a segment of MA is shown in **c**, with a micro ruler placed under the electrode to provide the scale.

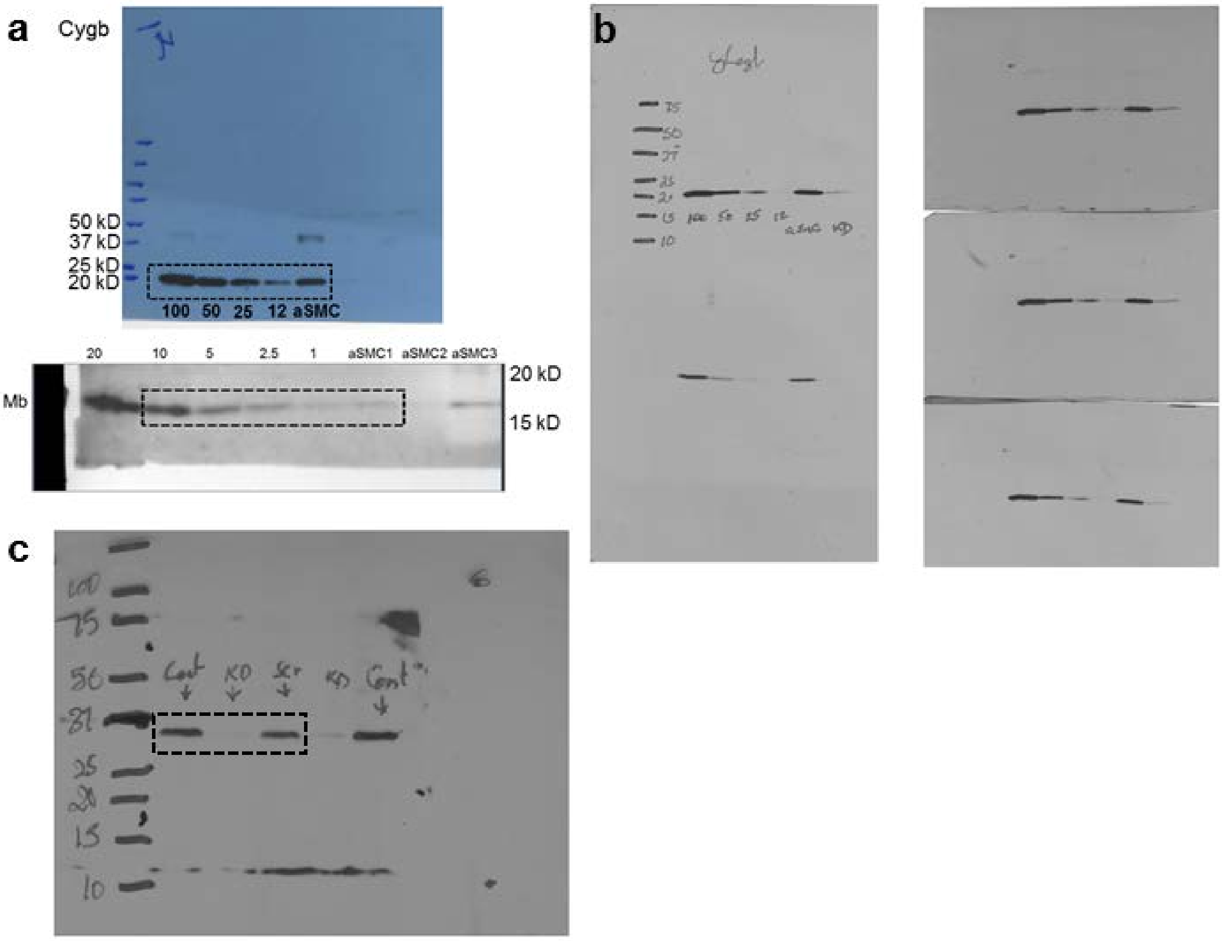

Supplementary Figure 4. Representative Western blots for the quantitative immunoblotting. **(a)** Western blots for cytoglobin and myoglobin as shown in Figure 1a and 1b. The top Western blot shows four lanes containing purified recombinant human cytoglobin at 100 ng, 50 ng, 25 ng and 12 ng, followed by the lysate extracted from rat aortic vascular smooth muscle cells (aSMC) and three lanes containing lysate from aSMCs treated with Cygb siRNA. The bottom Western blot shows five lanes containing myoglobin standards at 20 ng, 10 ng, 5 ng, 2.5 ng and 1 ng, followed by three lanes containing lysate from aSMCs. The bounding box shows the portion of the gel used in Figure 1 of the main text. **(b)** Representative Western blots for the quantitative immunoblotting of cytoglobin as shown in Figure 3 of the main text. Each blot shows four lanes containing purified recombinant human cytoglobin at 100 ng, 50 ng, 25 ng and 12 ng, followed by the lysate extracted from rat aortic vascular smooth muscle cells (aSMC) and cytoglobin siRNA-treated cells (KD). One Western blot is marked up to show approximate location of molecular weight markers, and the other four panels are repeats of the experiment. **(c)** Representative Western blot for the immunoblotting of cytochrome b5 reductase 3 as shown in Figure 4 of the main text. The five lanes contain purified recombinant human cytochrome b5 reductase 3 as control (lanes 1 and 5; Cont) and the lysates extracted from rat aortic smooth muscle cells treated with either cytochrome b5 reductase 3 siRNA (lanes 2 and 4; KD) or scramble siRNA (lane 3; Scr). The bounding box shows the portion of the gel used in Figure 4 of the main text.

References

1. Liu, X. & Zweier, J. L. Application of Electrode Methods in Studies of Nitric Oxide Metabolism and Diffusion Kinetics. *J Electroanal Chem (Lausanne Switz)* **688**, 32-39, doi:10.1016/j.jelechem.2012.09.038 (2013).
2. Xavier, F. E., Blanco-Rivero, J., Ferrer, M. & Balfagon, G. Endothelium modulates vasoconstrictor response to prostaglandin I2 in rat mesenteric resistance arteries: interaction between EP1 and TP receptors. *Br J Pharmacol* **158**, 1787-1795 (2009).
3. Thorin, E., Huang, P. L., Fishman, M. C. & Bevan, J. A. Nitric oxide inhibits alpha2-adrenoceptor-mediated endothelium-dependent vasodilation. *Circulation research* **82**, 1323-1329 (1998).
